# Traces of Archaic Mitochondrial Lineages Persist in Austronesian-Speaking Formosan Populations

**DOI:** 10.1371/journal.pbio.0030247

**Published:** 2005-07-05

**Authors:** Jean A Trejaut, Toomas Kivisild, Jun Hun Loo, Chien Liang Lee, Chun Lin He, Chia Jung Hsu, Zheng Yuan Li, Marie Lin

**Affiliations:** **1** Transfusion Medicine Laboratory, Mackay Memorial Hospital, Taipei, Taiwan,; **2** Estonian Biocentre, Tartu, Estonia; Massey UniversityNew Zealand

## Abstract

Genetic affinities between aboriginal Taiwanese and populations from Oceania and Southeast Asia have previously been explored through analyses of mitochondrial DNA (mtDNA), Y chromosomal DNA, and human leukocyte antigen loci. Recent genetic studies have supported the “slow boat” and “entangled bank” models according to which the Polynesian migration can be seen as an expansion from Melanesia without any major direct genetic thread leading back to its initiation from Taiwan. We assessed mtDNA variation in 640 individuals from nine tribes of the central mountain ranges and east coast regions of Taiwan. In contrast to the Han populations, the tribes showed a low frequency of haplogroups D4 and G, and an absence of haplogroups A, C, Z, M9, and M10. Also, more than 85% of the maternal lineages were nested within haplogroups B4, B5a, F1a, F3b, E, and M7. Although indicating a common origin of the populations of insular Southeast Asia and Oceania, most mtDNA lineages in Taiwanese aboriginal populations are grouped separately from those found in China and the Taiwan general (Han) population, suggesting a prevalence in the Taiwanese aboriginal gene pool of its initial late Pleistocene settlers. Interestingly, from complete mtDNA sequencing information, most B4a lineages were associated with three coding region substitutions, defining a new subclade, B4a1a, that endorses the origin of Polynesian migration from Taiwan. Coalescence times of B4a1a were 13.2 ± 3.8 thousand years (or 9.3 ± 2.5 thousand years in Papuans and Polynesians). Considering the lack of a common specific Y chromosomal element shared by the Taiwanese aboriginals and Polynesians, the mtDNA evidence provided here is also consistent with the suggestion that the proto-Oceanic societies would have been mainly matrilocal.

## Abstract 2

An extensive phylogenetic analysis of mtDNA from nine Taiwanese tribes reveals an unambiguous genetic link between aboriginal Taiwanese and Polynesian populations, to the exclusion of mainland Asians.

## Introduction

Present day Taiwan is a home to heterogeneous groups of people. The main part of the Taiwanese population is composed of the Minnan (73.5%) and Hakka (17.5%) who descend from immigrants from the Fujian and Guangdong provinces of Southeast China during the last 400 years. After World War II, migration from different provinces of China brought a present-day share of 7.5% to 13% to Taiwan [1]. Only 1.5% of today's population of Taiwan is represented by Austronesian speakers—Atayalic, East Formosan, Puyuma, Paiwan, Rukai, Tsouic, Bunun, Western plains, Northwest Formosan, and Malayo-Polynesian languages [2]—who are generally considered indigenous to the country. According to the year 2000 census, Yami with 4,050 individuals and Amis with 146,796 individuals represent the smallest and the largest tribal populations of Taiwan respectively.

The archaeological record suggests that a substantial cultural change occurred in Taiwan approximately in the sixth millennium BC [3]. Whether or not the transition to Neolithic technology in Taiwan corresponded to a substantial gene flow from China is unclear. It is also not clear whether there were one or multiple waves of Neolithic migrations to northern and southern Taiwan from southeast China [4–6]. There is archaeological evidence for the occupation of caves in southern Taiwan by humans by at least 15 thousand YBP [4]. Yet, the bulk of archaeological material started to accumulate during the Neolithic period.

Three different views have been put forward to explain the origin of present day Austronesian-speaking tribes in Taiwan, including (a) origin from expanding regions of early rice cultivation in central China [7], (b) origin in insular Southeast Asia or (c) local settlement after the rise of the sea levels [8]. During the massive immigration of Han speakers to the western plains of Taiwan, most tribes took refuge in remote regions such as the central mountain ranges or the east coast of Taiwan. It is believed that this geographical isolation has largely contributed toward maintaining their culture and languages until the present day in contrast to the plain tribes that are characterized by high levels of admixture [9].

The role of Taiwanese indigenous populations in the Austronesian settlement of island Southeast Asia and Polynesia has come under intense discussion during the last two decades, in particular among geneticists working with mitochondrial DNA (mtDNA), Y chromosome, and HLA loci [9–21]. Although the landscape of possible migration out of Taiwan and interaction scenarios is quite complex [22], two opposing models, the “express train” [23] and the “entangled bank” models [24], emerging from among other alternatives [8,16,25] have commonly been tested with genetic data. Proponents of the express train model [23] hold that the Polynesian islands were settled in a relatively short period with a migration originating from southern China to Taiwan. According to this model, the Austronesian migration from Taiwan was coupled by the spread of Lapita culture, whose supposed precursor pottery in Taiwan is dated to at least 6000 YBP [26]. The entangled bank model [24], along with the “slow boat” model [8,16,25], on the other hand, proposes that the ancestors of Polynesians did not move rapidly through Southeast Asia and Melanesia. According to these two models, the interaction with Melanesians before colonizing the Pacific was the major factor determining the genetic composition of the Polynesian ancestors. The proponents of the intermediary slow boat model [8,16,25], also popularly introduced in the book *Eden in the East* [27], propose a date and a location in Southeast Asia for the origin of the Polynesian expansion and question the Taiwanese genetic contribution to the ancestors of Polynesians.

Previous mtDNA studies [13,28] on four tribal groups (Amis, Atayal, Bunun, and Paiwan) and on the Taiwanese general population [12,29] have revealed their common ancestral origins with populations of China and/or Southeast Asia. Tajima et al. [9] characterized two hyper-variable segment I (HVS-I) lineage groups as unique to Taiwanese aboriginals, accounting for 22% of their mtDNA variation, and estimated their origin to approximately 11,000–26,000 years ago. Three groupings of aboriginal Taiwanese populations were suggested by this study [9]: southern (Rukai and Paiwan), northern (Atayal and Saisiat), and central-eastern (Amis, Bunun, Tsou, Puyuma, and Yami). However, the data did not conclusively show whether these three distinctive clusters reflected three different migrations to Taiwan or whether they could be explained by the effect of drift and long-term isolation of the island's populations.

A common HVS-I motif 16189–16217–16261, classified within haplogroup B4a [30], is shared between Taiwanese and Polynesian populations. This motif was considered first as the genetic link supporting Polynesian origins in Taiwan [10,13]. This root haplotype of B4a from which the Polynesian motif derives by a single HVS-I mutation is, however, widely spread in East Asia. Besides Taiwan and Southeast Asia, B4a occurs frequently in southern China and has been observed as far inland as among Mongolians [21,30–35]. Its deep time depth, 40,400 ± 9,600 years in China [34], significantly predates the timeframe that is relevant to the peopling of Polynesia. Therefore, this general genetic link between Taiwan and Polynesia has now been refuted as being supportive of Polynesian origins specifically in Taiwan within a recent timeframe as implicated by the fast train model [8].

**Figure 1:**
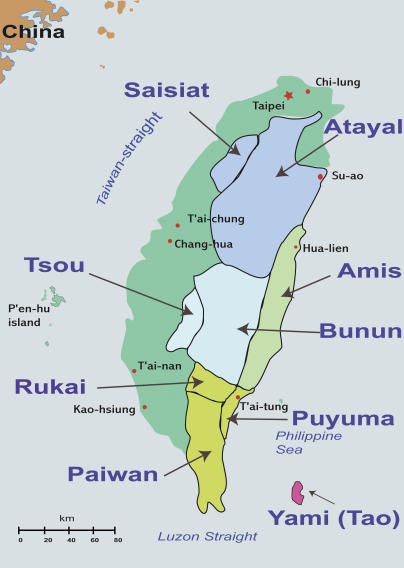
Geographic Distribution of Nine Indigenous Tribes of Taiwan

An overwhelming majority of mainland East Asian mtDNA lineages are nested within haplogroups A, G, M, N9, and R9 [30,31,34–38]. Although previous mtDNA research on Taiwanese aboriginal populations has focused only on hyper-variable displacement loop (D-loop) data, we attempt here to define the distribution of these haplogroups and their region-specific twigs in nine Taiwan indigenous tribes (Figure 1). Using the coalescent approach, we examine the hypothesis that the distinct mtDNA variation and genetic structure seen in present-day Taiwanese mountain tribes might be the result of a Neolithic migration or of a long-term separation from their common ancestors with the modern Chinese populations of mainland East Asia. Finally, to determine if Taiwanese B4a lineages share any coding region variants with Polynesian B4a lineages [39], we determined the complete mtDNA sequence for eight different B4a lineages from indigenous Taiwanese.

## Results

### Haplogroup Structure and Distribution in Taiwanese Aboriginal Populations

**Figure 2:**
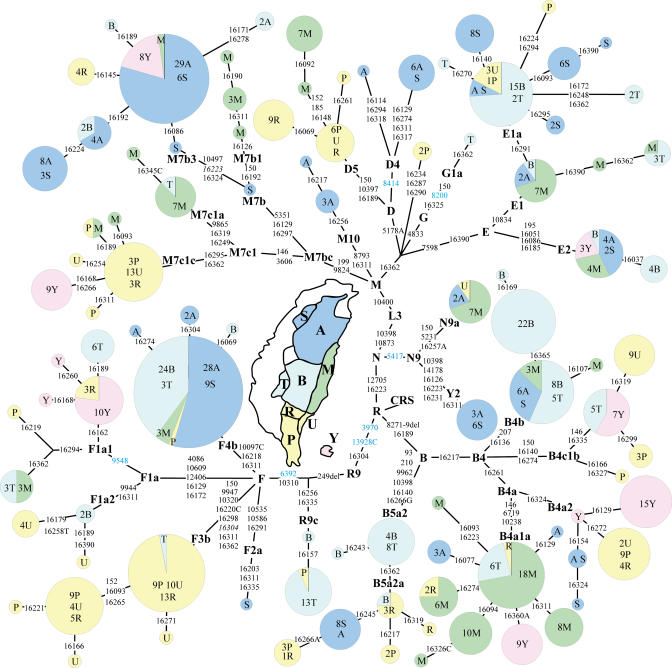
Tree Drawn from a Median-Joining Network of 96 mtDNA Haplotypes Observed in Nine Indigenous Taiwanese Populations The tree is based on sequences of HVS-I (16024–16390) and a coding region segment covering 9,793 to 10,899 bps. Haplogroup defining HVS-II mutations were manually added after the generation of the network. Additional coding region mutations, ascertained through complete mtDNA sequencing of an individual of each subclade of the haplogroup defined by the mutation, were used to generate the network and are shown in blue. All nps are numbered according to reference sequence [71]. Mutations in italic indicate back conversions. Nucleotide change is specified only for transversions. Node areas are proportional to haplotype frequencies of the pooled nine tribes. The population codes are as follows: A, Atayal; B, Bunun; M, Amis; P, Paiwan; R, Rukai; S, Saisiat; U, Puyuma; T, Tsou; and Y, Yami. CRS = Cambridge reference sequence [70].

Ninety-six distinct haplotypes were identified by 81 variable sites of the mtDNA control region in 640 Taiwanese aboriginal samples representing all nine mountain tribes (Figure S1). Among these, 39 individuals possessed a unique HVS-I haplotype. From the 96 haplotypes, 30 were shared by at least two different tribes. Haplotype sharing occurred mostly between adjacent tribes (Figures 2 and S1). Phylogenetic analysis using control and coding region information clustered the observed haplotypes into 20 distinct haplogroups and subgroups whose distribution among tribes was compared to available information from other Asian populations (Table 1). Four basic haplogroups—B, E, R9, and M7–accounted for more than 90% of the variation observed in aboriginal Taiwanese. In comparison, the combined frequency of these haplogroups in China averaged less than 40%, with southern Chinese having significantly higher frequency than northern Chinese (Table 1). Among these four basic haplogroups, haplogroup E was nearly absent in continental Asia. On the other hand, haplogroups A, D4, G, and M8-M10 accounted approximately for 41% of the sequences from the mainland, whereas it was rare or absent in Taiwanese Aborigines. Only two unique D4 haplotypes were observed in the Atayal and Saisiat populations and a single G1a lineage in Tsou (Table 1; Figures 2 and S1).

**Table 1:**
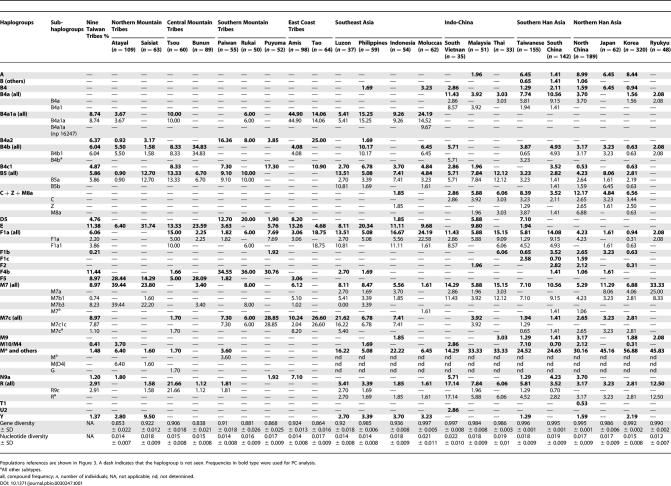
Haplogroup Frequencies in Taiwan, East Asia, and Oceania

### Principal Components Analysis

**Figure 3:**
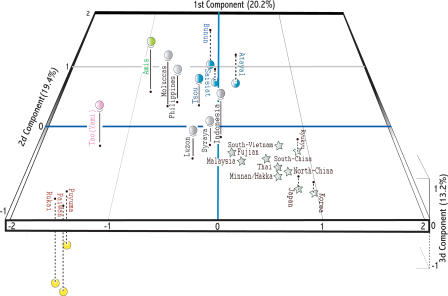
Principal Components Analysis Principal components map obtained from a matrix of haplogroup frequencies in nine Taiwan indigenous tribes (Table 1), northern and southern Chinese [34], Taiwan urban population (Minnan and Hakka) [29], Japan [73], Korea [33], Luzon [12], Moluccas [14], Indonesia, Ryukyu [42] Malaysia, South Vietnam, the Philippines [20], and Thai [45,46]. Austronesian-speaking populations are represented by circles, non–Austronesian-speaking populations by stars.

Principal components (PC) analysis using haplogroup frequencies revealed a high level of differentiation between Taiwanese aborigines as compared with other Asian populations (Figure 3). In particular, Taiwanese aboriginal populations appeared closer to island Southeast Asian populations (Luzon, Philippines, Moluccas, and Indonesia) than to populations from mainland East Asia (Fujian, South Vietnam, Malaysia, and Thailand). The three southernmost populations of Taiwan (Puyuma, Paiwan, and Rukai) and, more distantly, Yami from Orchid Island clearly differentiated the southern populations from the northern and central populations of Taiwan from which the Bunun sample emerged as an outlier. Although the Amis population clustered closely with northern and central tribes in the first two dimensions of the PC analysis, their haplogroup structure and relatively high frequency of B4a, D5, and M7c clades at the same time showed an affinity toward southern tribes of Taiwan. The grouping of Taiwanese indigenous populations revealed by our analysis differs from the population tree drawn from pairwise nucleotide differences by Tajima et al. [20] in which Puyuma and Yami, for example, clustered with central and eastern populations.

### Tribes of Northern and Central Taiwan

Haplogroups B4b, B5a2, E, F4b, and M7b covered more than 80% of the mtDNA variation observed in Atayal, Saisiat, and Bunun populations (north and central Taiwan; see Figure 2). The frequency of these haplogroups elsewhere in Taiwan was significantly lower (24%; *p* < 0.0001). Haplogroup E was most divergent and frequent in the Saisiat population in the northern mountain ranges of Taiwan.

Two subclades of haplogroup B5a can be defined on the basis of complete sequence information [37,38], available restriction fragment length polymorphism (RFLP) and HVS-I information from Southeast Asia [11,30,33,34,40,41], and our data: The loss of a *Hae*III site at nucleotide position (np) 6957 in association with 16266A allele defines the major subclade B5a1 spread in Southwest China, Thailand, Vietnam, and Nicobar Islands whereas the presence of +11146 *Dde*I site at np 11146 defines a B5a2 clade that is predominantly in association with 16266G allele. Within the B5a2 clade, available D-loop information allows us to postulate the presence of two further branches that are highly region specific in Southeast and East Asia: HVS-I motif 16140–16189–16266G-16362 (B5a2a), which so far has been observed exclusively in Taiwan [9,28], whereas another motif, 16140–16187–16189–16266A/G, characterizes all B5a variants observed so far in Korean, Japanese, and Han lineages from northeast China [33,34,40,42]. In Taiwanese aboriginals, B5a2 lineages were found all over the island whereas their frequency, likely due to drift, was the highest in north-central regions, among the Tsou and Saisiat (see Figure 2).

Over one quarter of Taiwanese from northern and central mountain regions (Atayal, Saisiat, and Bunun) belonged to a novel subclade of haplogroup F4 [38,39,43,44] called F4b because of its distinctive mutation motif 10097C-16218–16311 (see Figure 2). Considering available mtDNA datasets for Southeast Asia, haplogroup F4b has a marginally low frequency (<1%) in China [30,31,34] and was not found among 519 Thai samples [45,46], in which the overall haplogroup F frequency was one of the highest in Asia (22%).

Most northern Taiwanese aboriginals from the M7b clade possessed a combination of four HVS-I substitutions (at nps 16086–16129–16324), which defines the clade M7b3 [47]. HVS-I sequences with a similar motif have been observed previously only in one Hui, one Uighur, one Dai, and one Mongolian sequence [32,43,47,48]. Consistent with the study of Tajima et al. [9] in which the HVS-I–defined cluster C4 corresponds to our M7b3, we found no exact matches to the Taiwanese M7b3 sequences in the available literature. Two Taiwanese complete sequences with M7b3 HVS-I motif [39], one matching our haplotype 56, the other deriving from haplotype 60 by a single HVS-I mutation, shared seven coding region mutations (at nps 1664, 4454, 6351, 9468, 10497, 12121, and 14115) that have not been seen in other M7b sequences from China or Japan [37,38,49]. Furthermore, the Taiwanese M7b3 sequences lacked a mutation at np 12811 that is shared by sister clades M7b1 and M7b2 [37]. Interestingly, all Taiwanese aboriginal populations lacked the M7b2 subclade that occurs with remarkable haplotype diversity (*h* = 1) and frequency (8%) in neighboring Ryukyu island populations [42]. Also, subclade M7b1, which is common in southern Chinese [30] and the non-indigenous Taiwanese population (9% frequency; [42]) was found at low frequency only among the Amis. Interestingly, all five Amis M7b1 sequences derived from their ancestral haplotype by a substitution at np 16126 that was not observed among any other 28 M7b1 samples from Asia [30]. This observation makes unlikely the possibility that admixture with Han populations from China would be the source of the M7b1-16126C lineages seen in Amis.

Highly homogeneous haplogroup B4b lineages were concentrated in the northern and central regions of Taiwan, likely involving a founder effect in Bunun. This clade is frequent in southern China where an exact match for the dominant B4b haplotype is also observed among the Taiwanese [30].

Haplotype 30 (see Figure S1), observed in six Saisiat and three Atayal samples within haplogroup Y, included only a match with two Han Chinese from Shanghai [40]. Similarly, exact haplotype matches with Han Chinese from China were found among tribal samples grouped in haplogroups B4b and D4, which were observed only in the northern-central parts of Taiwan, in the Atayal, Saisiat, and Bunun populations. Haplogroups B4b and D4 could represent recent admixture mediated through urban populations. Nonetheless, this appears to be quite unlikely for the Bunun who have historically been one of the most isolated tribes in Taiwan, who practiced head hunting until the early 20th century. The high incidence of two specific B4b haplotypes among them, in contrast, can be well explained by drift operating in a strictly endogamous small community.

Thirteen Tsou sequences, one Paiwan, and one Bunun sample shared a characteristic haplogroup R9c substitution pattern [50] in the control region (see Figure 2). This haplogroup has been observed at low frequency in mainland Southeast Asia [34,50,51].

### Yami and Tribes of Southern and Southeastern Taiwan

Haplogroups B4a, D5, F3b, M7c, and N9a characterized 72.2% of the mtDNA variation of the populations of south and southeast Taiwan. Two distinct subclades of M7c were observed. The first subclade, M7c1c [30], occurs frequently all over the southeast coast, and is most frequent among Puyuma (29%). The second subclade, M7c1a, is seen in Amis (8%) and one Tsou sample. Haplotype diversity (*h* = 0.59) of M7c1 in Taiwan is likely reduced by genetic drift and founder effects among the Puyuma and Yami tribes. Similarly, without any downstream variation, only the founder HVS-I haplotype (16223–16257A) of haplogroup N9a was observed in Atayal, Amis, and Puyuma populations. Subclade B4c1b, frequent in southern Taiwan, has been previously detected at low frequency in China, Japan, Malaysia, Vietnam, the Philippines, and Java [13,20,30,38,51–53]. Two distinct subgroups of B4a can be found in Taiwanese aboriginals. The first of them, B4a1a, distinguished by a substitution at np 6719 (see Figures 2, S1), occurs frequently among the Amis and Yami, whereas the second, B4a2, is common in the southern tribes Paiwan, Puyuma, Rukai, and Yami.

Haplogroup F3b, previously labeled as R9a, has been encountered at low frequency in south and west China [30,34,37]. In Taiwanese, a high frequency of F3b was observed specifically among the three southernmost populations and is virtually absent in other tribes. This is consistent with the distribution of the respective HVS-I cluster C2 described in the study of Tajima et al. [9]. Interestingly, the HVS-I motif of all Taiwanese F3b samples was different from the motif commonly observed among the Chinese. Namely, Taiwanese F3b samples were characterized by a transversion at np 16220 (16220C) and lacked the transition at np 16335 seen in R9c. The 16220C variant of haplogroup F3b has been observed previously in two Bai samples from Yunnan Province of China and in one Japanese sample from Honshu [42,43]. In both cases, however, the difference with the closest Taiwanese HVS-I haplotype is two or more substitutions in HVS-I. Haplogroup F1a distribution is concentrated among the Tsou and Yami populations. Interestingly, among Yami, only the F1a1 subclade, defined by the HVS-I motif 16129–16162–16172–16304, was detected, although other haplogroup F subclades occur at considerably high frequency throughout other populations of Taiwan.

Finally, haplogroup D5 is spread at a moderately low frequency throughout East Asia, being most frequent in the south and absent or rare in Central Asia and Siberia [30]. Among Taiwanese aboriginal groups, distribution of D5 lineages is restricted to the three southernmost populations and the Amis. Although samples from Paiwan, Puyuma, and Rukai carry the root HVS-I haplotype of D5 or its one-step mutation descendants, the Amis harbor a derived motif with two substitutions (16148 and 16092) that has not been detected elsewhere in Asia so far.

### Coalescence Times

A number of mtDNA haplogroups in Taiwan showed limited haplotype and nucleotide diversity. Consequently, according to control sequence information, sequences belonging to haplogroups B4b, D4, E2, F4b, N9a, and Y (and possibly R9c when ignoring the unique Bunun specimen) coalesced in their most recent common ancestors (MRCAs) within the last two thousand years (Table 2). These low coalescence times may be due either to drift or to bottlenecks affecting locally evolving lineages, or they may be due to founder effects following admixture with recent immigrants from outside the island. The presence or absence of matching sequence types elsewhere, as detailed above, can be considered the best possible way to evaluate these two scenarios. The nearly complete absence of haplogroup E in China makes it unlikely that the shallow time depth of E2 lineages in Taiwan could be explained by a migration from China. However, many populations from island Southeast Asia are still poorly covered, and the possibility that these lineages can derive from a migration from outside should be kept open.

**Table 2:**
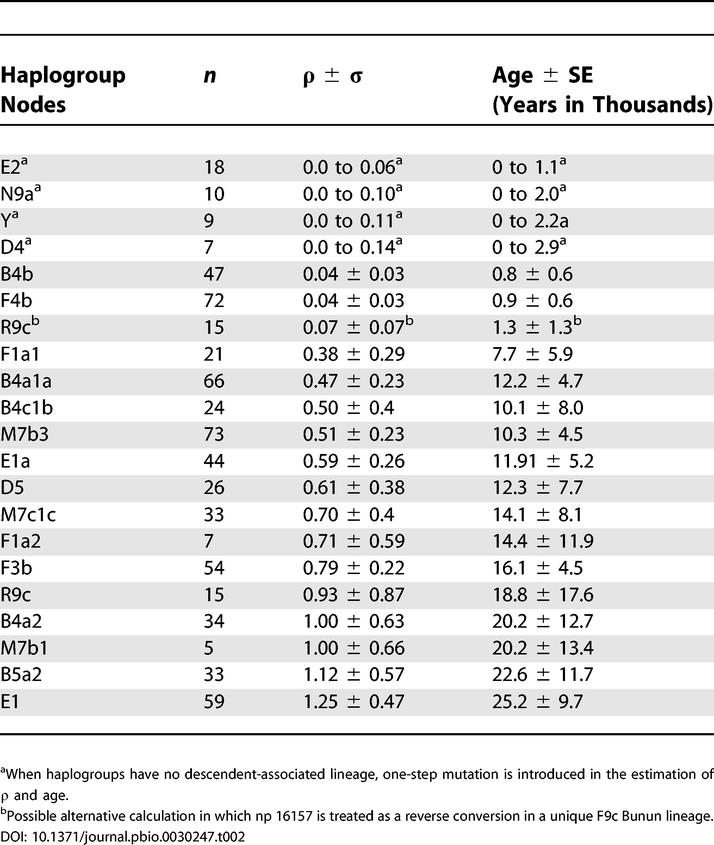
Coalescence Times of Major mtDNA Haplogroups in Taiwanese Aboriginals

More than half of Taiwanese mtDNA lineages fall into clades B4a1a, B4c1b, E1a, F1a1, F1a2, F3b, M7c1c, and M7b3 that show, with a broad range of standard errors, average coalescence times between 7.7 and 16.1 thousand years. The dates for clades M7c1c and M7b3 are similar to those of M7b1 and M7b2 previously reported in Southeast Asia [30]. It is likely that these four M7b daughter clades, together with other subclades of haplogroups B4, E, and F3b, began to diversify at the time of the rise of the sea levels after the end of the Younger Dryas cold spell approximately 11,000 years ago in distinct islands close to mainland Southeast Asia.

### Taiwanese Affinities with Austronesian Populations of Oceania

PC analysis reveals that Taiwanese populations in general and the Amis specifically are more closely related to island Southeast Asian populations than to populations from mainland East Asia (see Figure 2). However, only haplogroups B4a1a and M7c1c substantiate the close affinity between Taiwanese populations and those from islands beyond the Philippines. Characteristic HVS-I motifs of haplogroups B5a, F3b, R9c, and E2 were also observed in the Philippines and Sabah from Borneo; haplogroups Y and M7b3 were seen as shared only with Philippines' populations. Finally, F4b and R9c lineages are noncharacteristic of Near and Remote Oceania [11,12,20,39].

**Figure 4:**
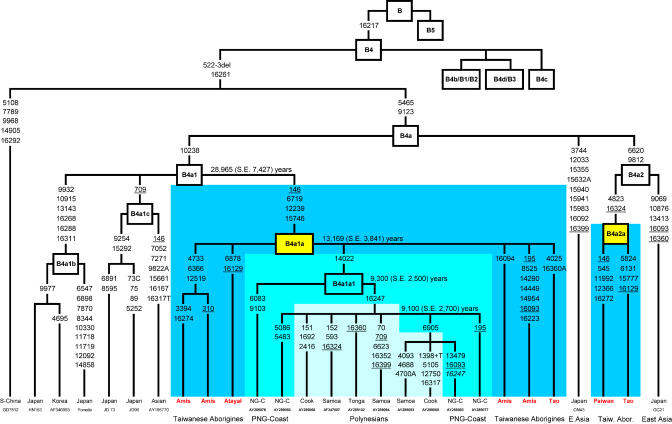
Phylogenetic Tree Relating Haplogroup B4a1 Complete Sequences Base pair exchange is specified only for transversions. Recurrent mutations are underlined. Coalescence times are shown beside nodes.

The B4a sequences, in general, are frequent along the southeastern coast of Taiwan, but also have a wide and ancient distribution all over East Asia and island Pacific. To provide a more detailed phylogenetic resolution, we determined the complete mtDNA sequence of eight different HVS-I haplotypes observed in Taiwan that belong to haplogroup B4a. Phylogenetic analysis, including 23 published B4a complete sequences, reveals an interesting substructure of the clade. One major subclade, B4a1, can be defined now by three coding region mutations at nps 5465, 9123, and 10238 (Figure 4) that cover all Taiwanese and Oceanian B4a sequences. The B4a clade also includes samples from Korea, Japan, and China. Using a mutation rate of 1.26 ± 0.08 × 10^−8^ in the mtDNA coding region [54], the coalescence time of B4a1 (28.9 ± 7.4 thousand years) is consistent with its origin, likely somewhere in Southeast Asia before the last glacial maximum. Notably, besides the B4a1 subclade, which occurs at minor frequency in Han Chinese (8/332; [30,34]), at least two other subclades of B4a can be found in southern China. Similarly, among Japanese, haplogroup B4a1 is represented by three different subclades that appear as sister groups to the Taiwanese/Polynesian-specific clade B4a1a (Figure 4). On the other hand, the minor B4a2 subclade among Taiwanese has one closely related lineage among Japanese.

The coalescence time of haplogroup B4a1a, based on coding region mutations, is 13.2 ± 3.8 thousand years, consistent with its HVS-I–based age estimate. To test whether B4a1a could have been imported recently to Taiwan from the mainland, we genotyped the defining markers of this clade in 47 North Vietnamese and 79 Han speakers from Fujian (unpublished data), the closest province of China to Taiwan, and we did not observe any carriers of haplogroup B4a1a. The clustering pattern within B4a1a therefore provides a unique link between Polynesian, Papuan, and Taiwanese lineages, supporting their common origin around the Younger Dryas period somewhere in island Southeast Asia, possibly in Taiwan. The Polynesian and Papuan sequences occupy a derived position in the B4a1a tree, descending from their MRCA defined by a mutation at np 14022 that was not observed in Taiwanese. The derived B4a1a1 clade in Papuans and Polynesians has a coalescence time of 9.3 ± 2.5 thousand years.

## Discussion

### Genetic Composition of mtDNA Lineages in Aboriginal Taiwanese

Analysis of the mtDNA haplogroup composition in aboriginal populations of Taiwan revealed highly distinctive patterns of the spread of the subgroups of common Asian specific haplogroups as compared to populations of China. On the one hand, haplogroups B, E, R9, and M7, which cover most of the lineages in aboriginal Taiwanese, have significantly lower frequencies in China. On the other hand, haplogroups A, C, G, and M8–M10, which are frequent in China, are completely absent in the nine tribes of Taiwan. In contrast to mainland China, the low haplotype diversity seen among small indigenous populations suggests that genetic drift may be responsible for generating the distinct frequency patterns reported in this study (Table 1).

Interestingly, however, haplogroups that make up the majority of lineages in Taiwanese aboriginals were also observed at high frequency (>60%; Table 1) among other island Southeast Asian populations who similarly present significant differences in their combined haplogroup frequencies with mainland East Asian populations. It is highly unlikely that the increment of the compound frequency of haplogroups B, E, R9, and M7 seen among all aboriginal populations of Taiwan could be explained by drift. It seems more plausible that the ancestral population of coastal East Asia and island Southeast Asia was already enriched by the founder lineages of these haplogroups and that drift affected the frequencies of individual haplogroups while their combined frequency throughout this region remained high.

In light of this study, the complete absence among aboriginal populations of several mtDNA haplogroups common on the Chinese mainland would suggest that the Neolithic colonizers (a) did not contribute significantly to the mtDNA pool of the pre-existing Formosan population; or (b) that the Neolithic migrants were a small group already lacking haplogroups A, C, G, D4, and M8–M10 that might have become frequent in southern China more recently.

Although possessing the same general haplogroup composition, the nine Taiwanese aboriginal populations are all significantly different from each other (in genetic distances), with southern populations showing a frequency pattern different from that of the central and northern groups (see Figure 3) and each population revealing its own specific founder haplotypes (see Figure 2). Given the low geographic distances between the sampling locations, this striking diversity between the tribes, seen also in HLA studies [18], may denote prevalence of strong inter-group cultural differences preserved through social isolation and endogamy of the tribes. Even though intermarriages may have been uncommon between the tribes, it is possible that small-scale admixture could have occurred through child adoption among the mountain tribes [55].

### Fitting the mtDNA Heritage of the Taiwanese Aboriginals into the Models of Austronesian Expansion

Genetic models explaining the origins of Austronesian migration can be categorized by the three following components: the place of origin, the time scale, and the correspondence with specific archaeological evidence. Studies based on Y chromosomal and mitochondrial markers have traced in Polynesians the presence of lineages that are characteristic of Melanesians and East Indonesians but which are absent in mainland East Asia and Taiwan [8,16,25,56]. Evidence for interaction or initiation of a human settlement process, directed toward Remote Oceania, somewhere in East Indonesia or Melanesia is also supported by the phylogenetic analyses of Polynesian rats, *Rattus exulans* [57]. Besides the Melanesian-specific component, both human mtDNA and Y chromosomal haplogroups found in Polynesians include those that are common in populations of mainland East Asia and also in Taiwan [22]. This general share of ancestry refers, first of all, to mtDNA haplogroup B4a, which is the most frequent lineage group among Polynesians. However, the specific HVS-I motif associated with Polynesian expansion occurs only in Near and Remote Oceania whereas its immediate ancestral sequence is common throughout East Asia and has a coalescence time significantly predating the Polynesian migration [21].

Phylogenetic analysis of complete mtDNA sequences (Figure 4) in this study reveals the presence of a motif of three coding region mutations (nps 6719, 12239, and 15746) that define haplogroup B4a1a and are shared among aboriginal Taiwanese, Melanesians, and Polynesians. No mainland East Asian population has yet been found to carry lineages derived from these three positions. This suggests that the motif may have evolved in populations living in or near Taiwan at the end of the Late Pleistocene period. Considering the differences between the Late Pleistocene and present-day shorelines of Southeast Asia, the B4a1 lineages may also have evolved in regions now submerged under the sea. As long as mtDNA lineages of the Philippines and Indonesia, in particular, have not been analyzed in similar detail, the question about the precise origin of B4a1a has to be left open. The presence of an additional coding region mutation (np 14022) that defines haplogroup B4a1a1 and the HVS-I transition at np 16247 in Melanesians and Polynesians points to a maturation phase of the Austronesian migration in East Indonesia or Melanesia, during which the final Polynesian motif of mutations was established.

Austronesian is the world's most widely distributed language family, yet nine of its ten subfamilies are restricted to Taiwanese aboriginal populations [2]. The phylogeny of B4a1a mitochondrial genomes resembles the linguistic reconstruction of Austronesian languages with five of its primary branches restricted to Taiwan and the sixth branch spread all over Oceania. The 9.3 ± 2.6 thousand-year-old coalescence date we obtained using only coding region information for B4a1a1 diversification in Papuans and Polynesians predates significantly the earliest signs of Lapita culture in the region (around 3,500 BP). Nonetheless, these findings provide the first direct phylogenetic evidence for the common ancestry of Austronesian and indigenous Taiwanese maternal lineages and their maturation phase in East Indonesia or Melanesia. High frequency of a tagging mitochondrial haplotype in contrast to the absence of such in the Y chromosomes of Polynesians and Taiwanese might lend credence to the suggestion that the proto-Austronesian communities were matriarchal [58] and matrilocal (as the Amis tribe still is in Taiwan) whereby the Y chromosome pool of the initial migrants was lost after being repeatedly diluted on the way toward Polynesia.

### Conclusions

Taiwanese aboriginal populations share their maternal ancestry with populations of mainland East Asia through haplogroups B, R9, and M7 as their main genetic components. At the same time the haplogroup structure at a finer phylogenetic resolution suggests relatively long-term isolation from the mainland populations. The coalescence times of B4a1a, F3b, F4b, R9c, and M7c1c lineages point to founder effects in Taiwan ranging from recent (0–2,000 years) to more ancient times (7,000–20,000 years). These results most likely reflect the drift in small endogamous populations of the island that became isolated by the rising sea levels after the last Ice Age.

The time element (13.2 ± 3.8 thousand years to the MRCA) obtained from the phylogenetic reconstruction of complete B4a1a sequences requires that we adopt a model according to which the origin of Austronesian migration can be traced back to Taiwan, and allows for the notion that it was followed by interaction periods elsewhere in Indonesia and finally in Melanesia where the complete motif specific to Polynesian B4a1a1 sequences (Polynesian motif) was developed.

## Materials and Methods

In this study the sequence variation of the mtDNA D-loop HVS-I region, nps 16006–16397 were characterized in 640 samples drawn from nine Taiwan indigenous mountain tribes representative of most languages, cultures, and geographical settlements seen on the island before the last four centuries. In addition, coding region (CR) nps 9959–10917 were sequenced and the mtDNA nine-bp deletion in the mtDNA intergenic region between the COII gene and the Lysine tRNA gene (COII/tRNA^LYS^) was screened by sequencing specific polymorphism (SSP) with primer pair L8215/H8297 [34]. Finally, significant RFLPs were used to remove unavoidable errors in haplogroup designation. The following tribes were selected: in the north of Taiwan, the Atayal (*n* = 109) and the Saisiat (*n* = 63); in the central mountain ranges, the Tsou (*n* = 60) and the Bunun (*n* = 89); in the east, the Amis (*n* = 98) and the Yami (*n* = 64); and in the south, the Rukai (*n* = 50), the Paiwan (*n* = 55), and the Puyuma (*n* = 52) (see Figure 1). All indigenous people had both parents belonging to the same tribe, and gave consent to participation in this study.

### DNA extraction

Blood samples were collected in ACD tubes and treated at the Transfusion Medicine Research Laboratory of the Mackay Memorial Hospital in Taipei. Genomic DNA was extracted from 500 μl of buffy coat using the QIAmp DNA kit (QIAml blood kit, Qiagen inc. Chatsworth, California, United States) with minor modifications to the procedure recommended by the manufacturer.

### DNA sequence analysis

Using primers L15997 and H16401 [59], a segment of 404 nucleotides from the D-loop HVS-I of the mtDNA was obtained. Primer pairs 5, 7, 8, 11–14, 19, and 24 F&R described in Rieder et al. [60] were used for typing informative RFLP sites using the following restriction enzymes: *Alu*I (nps 5176 and 13262), *Bsp*EI (np 4710), *Hae*III (np 663, 3391, and 8391), *Hha*I (nps 4830, 7598, and 9053), *Hinf*I (np 9820), *Hpa*I (np 12405), *Mbo*II (np 12704), and *Tsp*509I (np 5416) [34,61]. Primers 15F [60] and H10917 (5′ g AACAg CTAAATAggTTg 3′) were used to amplify a segment of 959 nucleotides used for sequencing both CR strands (nps 9959 to 10917). Length variation in the COII/tRNA^LYS^ (nine-bp deletion) region was assayed by PCR-SSP method, using primer pair L8215/H3274 [34]. PCR was performed in 20 μl reactions and carried out for 36 cycles using the following final conditions: 20 mM Tris-HCl (pH 8.4), 50 mM KCL, 0.2 mM deoxyribonucleotide triphosphate each, 2 mM MgCl_2_ , 0.25 U recombinant Taq DNA polymerase (Life Technologies, Taiwan), 5% glycerol, cresol red 0.075 mg/ml, 0.2 mM of each primer, and 30–100 ng of DNA sample. Thermal cycling conditions were 1 min. at 95 °C followed by 36 cycles of 95 °C for 10 s, 60 °C for 30 s, and 72 °C for 30 s. Following the thermal cycling, the samples were maintained at 75 °C for 10 min. Prior to sequencing, the amplified products were separated from excess dNTP and primers by first pre-treating with shrimp alkaline phosphatase (Sap) and Exo I enzymes (USB Product number US 70995 pre-sequencing Kit, Pharmacia, Taiwan) following the conditions recommended by the manufacturer (37 °C for 30 min and 85 °C for 15 min). For each individual, sequencing of nps 16006 to 16397 was performed on both strands using the Perkin-Elmer/Applied Biosystems Division (ABI Taiwan) DyeDeoxy Terminator Cycle Sequencing Kit (Foster City, California, United States) according to the recommendations of the manufacturer. Purification on a G50 sephadex column was performed before the final run on an automated DNA sequencer (ABI Model 377). In our inter-population analysis with other studies, the region including nps 16182 to 16183 was excluded because an upstream adenosine homo-polymeric region may cause poor sequencing. Consequently substitutions at nps 16182 and 16183 were not indicated in Figure 2.

### Data analysis

Indices of molecular variance were calculated in ARLEQUIN package 2.0 [62]. A neighbor-joining tree was constructed with 1,000 bootstrap replicas using MEGA2 package [63]. A PC map was constructed with ADE-4 package [64–66] using HVS-I haplogroups frequencies obtained from the nine indigenous tribes of Taiwan and other regions of Asia shown in table 1. Coalescence times were calculated using the ρ statistic and HVS-I mutation rate of one transition per 20,180 years in bps 16090–16365 [67,68]. For complete coding region sequences, a mutation rate of 1.7 × 10^−8^ substitutions per site per million years was used assuming 5 million years for the split between human and chimpanzee lineages [69]. A more conservative rate calibration 1.26 × 10^−8^ [54], assuming a 6.5 million-year-old split of human and chimpanzee mtDNA lineages would imply that all the coalescence times, based on complete sequence data, that are discussed in the text would be approximately 1.3 times older.

## Supporting Information

Figure S1 Ninety-Six Haplotypes Seen in 640 Taiwan Indigenous Individuals For HVS-I, HVS II, and nps 9793−10899, sequences are numbered according to the revised reference sequence [70,71]. Only transversions are specified and the number in bracket indicates that only this region has been sequenced. Restriction enzymes have sometimes been used instead of sequencing to infer nps 9824, 10238, 10310, 10320, 10397, 10398, and 10400 (HinfI, HphI, BspMI, Tsp509I, BsrI, DdeI, and AluI, respectively). Significant diagnostic restriction enzymes are indicated at the top of each RFLP column with losses or gains of RFLP sites shown as plus or minus “9-bp del” refers to the length variation between nps 8271 and 8280 in the COII/tRNA^LYS^ region as assayed by PCR-SSP method [34] with 1 denoting presence of the nine-bp deletion and 2 denoting non-deletion. The colored columns represent lineage distribution among populations; colors are region specific and are conserved in Figure 2. The minimum evolution tree was constructed with Mega2 [63] using Tamura-Nei pairwise distances [72]. Specimens sequenced from 9982 to 10838 only are annotated with a number sign.
(2.9 MB TIF).

### Accession Numbers

The GenBank accession numbers (http://www.ebi.ac.uk/embl/index.html) for HVS-I and HVS-II data in this article are as follows: HVS-I (AJ967036–AJ967675) and HVS-II (AJ967676–AJ968315). Complete sequence data: accession numbers AJ842744–AJ842751.

## Abbreviations

COII/tRNA^LYS^: mtDNA intergenic region between the COII gene and the Lysine tRNA gene
D-loop: displacement loop
HLA: human leukocyte antigen
HVS-I: hyper-variable segment I
MRCA: most recent common ancestor
mtDNA: mitochondrial DNA
np: nucleotide position
PC: principal components
RFLP: restriction fragment length polymorphism
