# Correction: Traces of Archaic Mitochondrial Lineages Persist in Austronesian-Speaking Formosan Populations

**DOI:** 10.1371/journal.pbio.0030376

**Published:** 2005-10-11

**Authors:** Jean A Trejaut, Toomas Kivisild, Jun Hun Loo, Chien Liang Lee, Chun Lin He, Chia Jung Hsu, Zheng Yuan Lee, Marie Lin

In *PLoS Biology*, volume 3, issue 8: DOI: 10.1371/journal.pbio.0030247

The seventh author's name was originally misspelled as Zheng Yuan Li; it should be Zheng Yuan Lee.

The first sentence of the Materials and Methods should read as follows: “In this study the sequence variation of the mtDNA D-loop HVS-I and HVS-II regions, nps 16006 to 16397 and nps 53 to 404, respectively, was analyzed in 640 samples drawn from nine Taiwan indigenous mountain tribes representative of most languages, cultures, and geographical settlements seen on the island before the last four centuries.”

The first two sentences in the section “Data Sequence Analysis” in the Materials and Methods should read as follows: “Using primer pairs L15997–H16401 and L048–H408 [59], segments of 404 bps and 360 bps from the D-loop HVS-I and HVS-II, respectively, were obtained. Primer pairs 5, 7, 8, 11–14, 19, and 24 F&R described in Rieder et al. [60]….”

